# ‘’We usually choose safety over freedom’’: results from focus groups with professional caregivers in long-term dementia care

**DOI:** 10.1186/s12913-022-07952-0

**Published:** 2022-05-20

**Authors:** Suzanne Portegijs, Adriana Petronella Anna van Beek, Lilian Huibertina Davida van Tuyl, Cordula Wagner

**Affiliations:** 1 Netherlands Institute for Health Services Research (Nivel), PO Box 1568, 3513 CR Utrecht, The Netherlands; 2 Department of Public and Occupational Health, Amsterdam UMC, Amsterdam Public Health Research Institute (APH), Vrije Universiteit Amsterdam, Van der Boechorststraat 7, 1081 BT Amsterdam, The Netherlands

**Keywords:** Dementia, Long-term care, Physical activity, Group dynamic, Professional caregivers, Safety

## Abstract

**Background:**

People with dementia living in nursing homes are mostly sedentary, which is a consequence of various personal, environmental and organizational factors. Until now, studies on physical activity and safety in dementia have focused on residents and caregivers from the viewpoint of (individual) care provision and health benefits. There has been little to no focus on the possible influence of group dynamics between care providers with regard to physical activity and safety. The aim of this study is to gain more insight into the viewpoints and intentions of groups of professional caregivers towards safety and physical activity and the potential influence of the group-oriented setting in long-term care on physical activity of individual residents.

**Methods:**

A qualitative study comprising three focus group discussions including professional caregivers (*n* = 15) was conducted within two long-term care organizations in the Netherlands. Focus group discussions were structured using an interview guide derived from a preliminary framework, based on existing literature and complemented with clinical expertise.

**Results:**

Seven themes could be derived from the focus group discussions that influence physical activity and safety: 1) Individual health and abilities; 2) Balancing physical activity and safety; 3) Physical restraints; 4) Group interests versus the individual interests; 5) Organization of care and physical environment; 6) Perceived responsibilities and tasks of professional caregivers and 7) Change is challenging.

**Conclusions:**

Due to multiple influencing factors, the balance for care providers in long-term care generally tends towards safety over physical activity. Furthermore, in order to stimulate physical activity various limitations are experienced, including the organization of care, the general health of the residents and difficulty to achieve changes in daily care. Most importantly, the group interests of both the professional caregivers and the residents have a substantial influence on the incorporation of physical activity in daily care.

**Supplementary Information:**

The online version contains supplementary material available at 10.1186/s12913-022-07952-0.

## Introduction

Physical activity positively influences multiple health outcomes of people with dementia (living in long-term care facilities), including cognitive functioning, independency in activities of daily living, physical performance, mobility, depression, agitation and functional ability [1–4]. Despite the positive effects, physical activity is generally low in nursing home residents [5–9]. This is a consequence of numerous influential factors such as the environment’s compatibility with the abilities of a resident, accessibility, security and comfort [10]. Also, the physical health of the residents, cognitive functioning and perceived feeling of understanding and personal freedom are imperative for the degree of physical activity among residents with dementia [11, 12].

When it comes to improving the degree of physical activity, care teams of nursing staff in long-term care facilities are a crucial influencing factor, as they spend the largest amount of time with residents compared to other caregivers involved [13]. However, various organizational factors impede the improvement of physical activity including staffing levels, available time and the amount and type of care provided [10, 11]. Due to insufficient staffing levels and/or (perceived) limited amount of time, professional caregivers are tended to use short cuts to meet basic care needs. Commonly at the expense of physical exercise and/or functional activities [14–16]. Furthermore, lack of clinical and administrative support, insufficient communication and team effort, concerns regarding anxiety and agitated behaviour of residents and injury for both residents and professional caregivers are also imperative barriers [12, 15, 17]. Despite the experienced barriers, most professional caregivers are conscious of the benefits of physical activity and state that it is feasible to improve the degree of physical activity in daily long-term care [14, 18].

The composition of care teams and organization of care within long-term care facilities is unique in comparison to other health care settings due to the educationally diverse care teams including registered nurses, Certified Nursing Assistants (CNAs) and assistants without a healthcare-related education [19–22]. While scientific studies on this topic present varying results, a diverse mix of caregivers and skills within a supportive context seems to have a positive influence on the quality of care [21, 23, 24].

Currently, the existing body of literature on the influence of group dynamics on long-term care is scarce and broadly focussed [19, 25]. However, from current studies and clinical practice it is evident that the organization of long-term institutionalized (dementia) care is mostly provided on closed wards [26] and largely group-oriented for both residents and professional caregivers [20, 22, 27]. The group-orientation for residents is mainly characterized by the majority of their time spend in shared spaces or ‘living rooms’ where the meals are served at set times and leisure activities are undertaken that fit the majority of the group [24]. Furthermore, daily care provided by the professional caregivers has a group character due to their shared responsibility for the care tasks to be performed [20, 22] and close proximity to each other.

Even though research on group dynamic within long-term care facilities is limited, available studies in general show that socially cohesive care teams positively influence job and organizational performance, job satisfaction and quality of care [19, 25, 28–32]. Yet, scientific studies on the views of groups of professional caregivers towards physical activity and the potential influence of the group-oriented setting in long-term care are lacking.

## Methods

### Aim

The aim of this study is to gain more insight into the viewpoints and intentions of groups of professional caregivers towards safety and physical activity and the potential influence of the group-oriented setting in long-term care on physical activity of individual residents.

### Reporting guidelines

In order to ensure an accurate description of the methods followed within this study, the Consolidated criteria for reporting qualitative research (COREQ) was used [33]. This checklist consists of 32-items reflecting three important domains regarding qualitative research, namely: research team and reflexivity, study design and analysis and findings.

### Design

This study is part of a larger research project in collaboration with a care organization in the Netherlands that wants to improve the living conditions of people with dementia within one of their nursing homes by providing more freedom and autonomy and facilitating social interaction with the surrounding neighbourhood. This care organization is included within this study. In addition, in order to gain a more broad view on this topic and include a more accurate reflection of the Dutch long-term care system, two additional nursing homes (both a large-scale and small-scale facility) were asked to participate. In these organizations, located in both a rural and urban area in the Netherlands, three focus groups were conducted in September 2019.

### Sample and invitation procedure

Nurses, Certified Nursing Assistants (CNAs), trainees and recreational coaches working at the long-term dementia care ward within the three included nursing homes were eligible for inclusion. All professional caregivers working on the ward were invited to participate in the focus group through an e-mail send by the care manager. Subsequently, the majority of the professional caregivers present on the specific day participated. A few (mostly two or three) professional caregivers remained at the ward to provide care for the residents. This resulted in a total of 15 participating professional caregivers. Group size varied between 3 and 7 participants and consisted of CNAs (*n* = 7), supplemented with recreational coaches (*n* = 2), nurses (*n* = 2), nurse in training (*n* = 1) and CNA trainees (*n* = 3). One participant was male and all others were female. The ages of the participants ranged from 22 to 64 and the years of experience in care from 0.5 up to 40.

### Setting

Two researchers were present at each focus group. The researchers introduced themselves before the start of the focus group and explained the aim of the research to the participants. The focus groups lasted for about one hour and were conducted around the change of shift in the afternoon as the largest number of professional caregivers is present at the wards around this time. Furthermore, the focus groups took place in a room on or near the wards and was conducted during work-time in order to lower the threshold to participate. All focus groups were audio recorded. After finishing the focus groups, the obtained data was discussed among the researchers. The initial plan for this study included three focus groups in total with the aim to achieve saturation. The researchers were able to discuss all topics adequately and many recurring themes were found within the focus groups.

### Interview guide

An interview guide was created based on literature review and expert opinion (see Appendix 1). In addition to the input obtained from the existing literature, expert opinion was obtained from both authors SP and AvB. Author SP is a physiotherapist with almost ten years of experience in long-term dementia care. Author AvB is a very experienced researcher and policy advisor within long-term care, specifically dementia care. Moreover, author CW is a professor in the field of patient safety and provided valuable insight and contributed meaningfully in the development of the preliminary framework and subsequent interview guide. The assumption within the preliminary framework is that some form of decision-making takes place between the degree of physical activity and safety by professional caregivers before they activate people with dementia. Another assumption is that the degree of physical activity and safety are influenced by both the professional caregivers and the residents.

### Data analyses

Transcripts were coded manually in Word. Subsequently, all quotes were categorized in Excel with a different tab for every individual code. The focus group discussions were analysed using a combination of deductive and inductive content analysis [34]. Firstly, a preliminary framework was created based on the interview guide to conduct deductive analyses of a-priori anticipated constructs. In parallel, inductive analysis included open and unstructured coding of constructs that did not fit into the a-priori constructs. Two researchers independently coded all transcripts (SP, AvB) and findings were discussed in weekly meetings.

The preliminary framework for deductive analysis included 10 codes. The inductive analysis added another 6 codes. This resulted in a final framework of 16 codes (see Table 1). Codes were subsequently grouped into themes by two independent researchers. All quotes that were not coded equally into one of the themes by the two researchers were discussed until consensus was reached. Quotes used within the article were translated from Dutch to English by an external native speaker and translated back by one of the researchers (LvT) and subsequently discussed by three researchers (SP, AvB and LvT) involved within this study to ensure an accurate translation.

**Table 1:** The initial ten codes and final 16 codes used during the analyses of the data

Initial ten codes	Final 16 codes
1. Residents	1. Resident
2. Quality of life	2. Well-being
3. Safety	3. Health
4. Physical activity	4. Activities
5. Independency in ADL	5. Safety
6. Autonomy	6. Physical activity
7. Decision-making	7. Independency in ADL
8. Professional caregivers	8. Autonomy/Freedom
9. Organization of care	9. Tools
10. Attitude/behaviour	10. Physical environment
	11. Outdoors
	12. Building
	13. Decision-making
	14. Professional caregivers
	15. Organization of care
	16. Attitude/behaviour

### Ethical considerations

This research has been conducted in accordance with the ethical standards of the Declaration of Helsinki, the Conduct Health Research (https://www.federa.org/codes-conduct) and the relevant guidelines regarding informed consent and the review of research ethics committees in the Netherlands. Furthermore, this study has been reviewed as part of the ZonMw dementia research and innovation program ‘Memorabel’, part of the ‘Deltaplan for Dementia’. Permission to conduct the research was provided by the location and care managers of the wards. Informed consent was obtained from all participants before the start of the focus group and recorded. All focus groups were recorded and transcribed verbatim. For privacy reasons, names of residents, professional caregivers, wards and care organizations were anonymized.

## Results

**Fig. 1:**
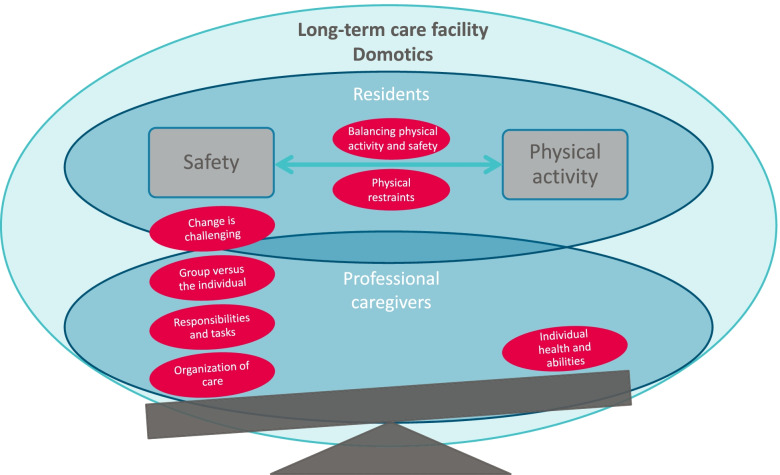
Aspects influencing the degree of physical activity and safety, within the context of long-term care facilities

Based on the 16 codes derived from the data seven themes could be identified, namely: 1) ‘Individual health and abilities’, 2) ‘Balancing physical activity and safety’, 3) ‘Physical restraints’, 4) ‘Group interests versus the individual interests’, 5) ‘Organization of care and physical environment’, 6) ‘Perceived responsibilities and tasks of professional caregivers’, 7) ‘Change is challenging’. In the following section, all themes are described and their interrelationship is reflected in Fig. 1.

### Individual health and abilities

Many different forms of physical activity are mentioned by the participants during the focus groups, including independency in- or assisting during daily care, meals and housekeeping, walking, swimming, group activities such as yoga or active games, dancing, drawing or painting and making music. In general, participants consider physical activity to be important due to its health benefits for residents such as preventing contractures, maintaining their independency and less uncomfortable daily care.

“The less they move, the stiffer they get you know. That’s unpleasant. Moving is pretty important..” [Certified nursing assistant]

Participants mention that there is a large variety among residents with regard to their independency in physical activity. Especially the lack of initiative among some residents is considered challenging.

“Well, some of our residents have a very strong urge to walk while with others we have trouble getting them to move. Uh. they sit, they walk from point A to point B and then sit down again until we tell them that we go to the room or the toilet. So the rest of the time, they don’t do much..” [Certified nursing assistant]

However, providing care that suits residents who tend to compulsively walk long distances every day also requires attention from the professional caregivers.

“We try to use the walker and every now and then, we give it to them. But sometimes… eh.. we are short staffed and we don’t always see everything so that lady fell for some reason… eh.. Sometimes we have people walking around all the time, then you have to supervise all the time and be alert because when they get exhausted they might fall. Some people have an urge to keep walking and we can’t calm them down.. at some point they get all tired and are more at risk of falling down.“ [Certified nursing assistant]

Furthermore, the degree of physical activity and the ability to assist during daily life activities differs among the residents.

“Because on 6 [red: floor 6 of nursing home] we have a gentleman, well he loves to help. We will give him a cloth and he instantly starts to clean the tables. He loves it when we ask him if he wants to go downstairs to help do grocery shopping, another lady loves to help too. She doesn’t like it when we make coffee for her, she likes to help with the laundry. Every person is different that way.“ [Certified nursing assistant]

Maintaining independency is considered challenging. In order to prevent functional decline, constant maintenance and stimulating physical activity is necessary for some residents.

“My opinion is that they stay mobile as long as they keep moving. When we let them sit in chairs then they.. eh.. in no time they will experience difficulty moving, much more passive. We have someone in room 43 who we had to medicate for her behaviour and she slept all the time because of it. Within two weeks she stopped walking. We’re talking about two weeks. That’s how fast it can happen.” [Certified nursing assistant]

“They get hospitalized and thus get spoiled. So at some point, when they get dressed by someone else all the time, they tend to stop helping or dressing themselves at all. So you have to let people keep doing their own things as long as possible.” [Nurse in training]

Alternative ways to stimulate physical activity are mentioned in order to make it more attractive for residents and provide a more diverse program.

“A.. eh.. pillow fight, I think this is a way you can let them move without them knowing. Because it’s a game they move more then they know.” [Recreational coach]

During the focus groups the influence of residents on daily care, safety and physical activity is mentioned to be of importance. One aspect that influences the degree of physical activity is their general health and physical abilities. A poorer health and/or physical ability influences physical activity in a negative way.

“The eh… general deterioration. It just simply doesn’t eh… for example, when they have a fractured bone and they have to recover and be rehabilitated and they usually don’t get out of their wheelchair or even their bed anymore.” [Nurse in training]

Furthermore, tailoring activities to the residents’ interests and hobbies is also mentioned to be very important to stimulate more physical activity.

“I make… I usually make some sort of vague image… then he.. he..he is fully awake. He (name of resident) looks at me and I ask ‘What do you think? Does it need any more work?’ He tells me that my perspective is pretty good.. but this area needs work. He goes on to tell me about all different kinds of painting techniques and how to preserve your paint. Everything is sorted by colour… then he laughs and starts to make jokes.. then he sits upright, he shuffles to the front of his seat… It doesn’t have to represent anything, it doesn’t matter.” [Recreational coach]

Providing and organizing activities that suit the interests of residents is found to have a positive influence on their general well-being. Alongside with a tailored approach for every individual resident during daily care.

“Just some of those little activities that you can do on a daily basis. There’s something to do every day. Sometimes you just put the resident in a room and give them a puzzle. You just watch and see what they do with it. Or maybe someone loves to do math, then you give them some equations to do.” [Certified nursing assistant]

“Yes… eh…. I love to use humour in my work, when I think it’s appropriate of course… Some resident have a need for it. There’s this man who lets us know that he likes it when we use humour. He said that he has a low mood, but this helps him…. Happiness and cozyness. “ [Certified nursing assistant]

Lastly, the importance of maintaining the identity of the individual person is mentioned to be a part of daily care provided by professional caregivers.

“She’s just being herself. That’s what I like, people just being themselves. Despite of them forgetting, sometimes we can help them find themselves and to love the things they do and experience. Authenticity.“ [Recreational coach]

### Balancing physical activity and safety

Within the previous theme, the benefits of physical activity are clearly stated by the participants. Though, they also experience safety concerns and distinguish two points of view: their own safety and the safety of the residents. With regard to their own safety, there are certain conditions the ward should meet in order to create a safe environment for the professional caregivers. For instance locking all the cabinets, the cutlery drawer, making sure no sharp objects are laying around and not feeling threatened by residents. Especially during certain time periods during the day or night when there is less staff present on the ward.

“It used to be different. There are moments where we eh.. mostly during the night. We only have two persons working at the same time. They have felt really threatened. At that moment, when there is a new resident coming in, who was feeling uncomfortable and then for instance chases personnel with a metal bar… Those moments are not safe, luckily this doesn’t happen very often.” [Nurse in training]

Although they do mention the occurrence of threatening situations or aggressive behaviour, these situations are rare and overall they feel safe.

With regard to the safety of residents, various physical conditions are mentioned: no wet floors, not fully opening windows, having sufficient fire prevention and having well-working tools such as wheelchairs and walking aids. Furthermore, the necessity to know the residents is mentioned in order to provide a safe environment and being able to address their needs. Despite the awareness for safety, participants mention they are not always able to provide a fully safe environment.

“We try to cover everything, to make sure that we give every patient what we think they need. Despite of doing that, we can’t be 100% sure that everyone gets what they need.” [Nurse]

A major concern with regard to the safety of residents mentioned by the participants is the risk of falling and their efforts to prevent that from happening. Perspectives and views on this topic vary among the participants. Some participants mention that safety and fall prevention is more important than providing autonomy for the residents and should be the main focus of care.

“Of course we have a lot of patients that walk very badly, even with a walker. So we have to watch them all the time, we keep giving them the walker and make sure they don’t walk unsupervised by themselves.” [Certified nursing assistant]

While others argue that almost all residents are at a higher risk of falling and that it is impossible to provide a fully safe environment.

“For example, we have patients who walk all the time but are at risk of falling. We can’t supervise them 24/7, you know. “ [Certified nursing assistant]

Some participants mention that they are torn between wanting to protect the residents, making sure they are safe and prevent falls, and the benefits the residents have by having more autonomy and physical activity in their daily life.

“Well, sometimes eh.. sometimes we just let them walk and they eventually fall. Then we take care of the wounds.. eh.. they still walk.. why would we limit them?” [Nurse in training]

“I think that’s it.. the freedom we can give these people. The freedom that they can choose to walk around, which we try to give them that as much as possible, of course as safe as possible. That’s the only thing that comes to mind. The degree of freedom that is still possible, we want to give that to them.” [Recreational coach]

### Physical restraints

The use of physical restraints in order to prevent falling and provide safety for the residents is mentioned by the participants. The main focus is on bed railings, fall mats and sensors. However, some also mention other physical restraints, such as sedating medications and the use of a Global Positioning System (GPS). Most participants comment that physical restraints are not used often in order to limit the residents, but to address their own needs and provide support and assistance. For instance: bed railings are fully up during the night at a residents’ own request or using a sensor during the evenings to be able to assist residents to the toilet. Participants also mention the possible health disadvantages that the use of physical restraints can cause and state that physical activity has a higher priority.

“With agitated patients we don’t use medication very often. Movement, getting them to move, is the most important for us. Because when you give them medication they tend to fall a lot more, that has a lot of consequences.“ [Certified nursing assistant]

### Group interests versus the individual interests

Balancing the need of the individual and the larger group is challenging for professional caregivers and makes them face ethical dilemmas with regard to safety and physical activity in daily care. Despite the focus on the needs of the individual, the solution is usually based on the interests of the group.

“Yes, we used to have a resident that couldn’t go downstairs to join the activities because of the risk of running away. So.. this was discussed and we also wrote it down in the treatment plan. He couldn’t participate in any of the activities. It’s sad, but.. if he would run away…” [Certified nursing assistant]

“What I sometimes find difficult is… there’s this one lady and she really wants to go outside, but I know she doesn’t want to come back inside. So for her own safety and the safety of the other people I take outside, it’s a problem. I feel this is an ethical dilemma. Should I refuse to take her outside so that I don’t endanger the group?“ [Recreational coach]

Participants also face safety challenges with regard to their colleagues when organizing more individual activities for residents. They feel like they cannot leave other professional caregivers behind at the ward due to safety issues. Subsequently resulting in that the interests of the group take precedence over that of the individual.

“And because of this it is limiting people to move. Or just to walk around the block. That’s not possible. Not in this department. Because of the types of residents we have here, you can’t leave your colleagues alone even with the two of them.” [Certified nursing assistant]

### Organization of care and physical environment

A subject that is discussed a lot during the focus groups is the influence of the organization of care on the degree of physical activity. Mainly sufficient staffing levels on the wards is mentioned to be of great importance in order to stimulate physical activity and independence during daily care.

“Eh..yes.. eh.. what you said.. so for example.. eh.. someone is putting on their pants really slowly.. then I would eh.. But what do you do when you are understaffed? So eh.. we quickly put on the pants, the pants and the inco [red: incontinence garments], and we let him finish the rest himself. So we helped a little bit so he can hurry up.” [Certified nursing assistant]

Also the organization of activities on the wards is considered challenging due to lower staffing levels.

“I don’t think we are the only one with a problem, I think a lot of nursing homes encounter the same things. The budget cuts are the cause of us being understaffed and too busy to do certain things with our residents. That’s a shame. We work all day until the evening shift comes in, then we can breathe again.” [Certified nursing assistant]

“Of course there are activities, and we do have quite days with sufficient staff, but it just happens too often that the staffing levels is not arranged well, or cannot be arranged, which makes that you can’t do many things with elderly, things we were able to do in the past.” [Certified nursing assistant]

The inclusion of multiple disciplines is typical for the Dutch long-term care and is considered valuable by the professional caregivers. For instance by having hosts and/or nutrition assistants during the meals and evenings to support the professional caregivers. Working with multiple disciplines is not only mentioned valuable for stimulating more physical activity, but also for daily decision making with regard to the care provided. For instance by consulting a psychologist if a residents shows challenging behaviour.

“So eh… we often consult the psychologist, you know. So first we observe and then we report on the matter. And then we gather our team together and she (the psychologist) gives us advise. Sometimes really small things can agitate a patient. So we try and we try. But sometimes the doctor has to take action, sometimes after all this trying we just don’t know.“ [Nurse]

Or for determining whether physical restraints need to be used in the daily care of a resident.

“Somethings… somethings are also physical restraints. So you are not allowed to put the bed railings up.. those types of things. We really have to ask the doctor about this.. they make the decision and after that we can perform certain restrictions.“ [Certified nursing assistant]

Some participants state that due to their lower staffing levels they are not able at all to stimulate physical activity or organize activities for the residents on the wards and need support from either activity counsellors, volunteers, physiotherapists or family members.

“So we tell them; If you want more, you have to come here and do it yourself. You have to take him out, walking, every day. We can’t do that.. we don’t have the staff available to do that.“ [Nurse in training]

The benefits of going outside for residents’ well-being is mentioned by the majority of the participants.

“Oh, I would love to go outside. When the weather is nice, going outside would be wonderful. They would love it, they really enjoy it.” [Certified nursing assistant]

Furthermore, multiple aspects with regard to the inner physical environment of long-term care wards for people with dementia are mentioned by the participants. Such as having a separate space where larger group activities can be organized. Furthermore, sufficient walking space, making sure no physical barriers on the wards are visible for residents, providing seating areas and interactive corners which draws the attention of the residents are considered important to reduce challenging behaviour.

“In their experience, that happens a lot. When people have the feeling they can go somewhere, that they’re not being restricted… then the agitation is gone.” [Certified nursing assistant]

Lastly, some participants mention that a ward that gives residents the opportunity to go outside independently and walk unobstructed has a positive influence on challenging behaviour.

“That’s our advantage. That our halls are pretty wide, and our building is a bit bigger. So people can walk more rounds outside. There are certain people that walk a marathon every day. So we oblige by that. You could eh…. Eh.. the only movement they get is in our department and they can move freely there. If they want to go outside, someone should join them. So family or caretakers, sometimes they take them outside. So.. a lot of movement?... No.” [Certified nursing assistant]

### Perceived responsibilities and tasks of professional caregivers

Some participants state that organizing activities and stimulating physical activity is not their responsibility or duty.

“For example going outside in a group. Now they only get to be inside. In the past they got to do that more often. Or there used to be more exercise inside. With activities. That is happening less than before. That’s a shame. But we can’t do anything about it.“ [Certified nursing assistant]

Similar statements are made with regard to the use of physical restraints. Participants seem to transfer the full responsibility for the use of physical restraints towards the geriatric specialists and legal authorities and claim that physical restraints provide safety for residents.

“We don’t do that. The doctor does it. The doctor makes the decision to limit their freedom. Because the restrictions of their freedom are for their own safety.” [Nurse in training]

On the contrary, the participants are aware of the laws and regulations on the use of physical restraints and mention that that is partially the reason they favour physical activity over safety.

“If someone keeps walking we can’t stop them. You can’t tie people up, so what are we supposed to do? So we tend to put freedom over safety.” [Certified nursing assistant]

Lastly, the collaboration between professional caregivers and being flexible with the daily time schedule is also mentioned to be important in order to have sufficient time for daily care and to stimulate physical activity.

“Yes, with some co-workers I work differently than with others. You just know; this is how we do things. So you could work with someone who really takes their time with the patients, and I would be the one who would like to do things quickly. That might clash. I usually think to myself; I can’t fight time. So when I start at 7 AM and I work until 4 PM and I have to help someone to get out of bed at 2:30 PM.. so be it. 4 PM is my time to go. But if you keep your calm and prioritize you get it done. So this is how I work; I have a unit where I have to wash someone in bed and I have to feed people, I usually start doing that so that everyone has eaten. So that’s one thing I will never forget, that everyone has eaten and is drinking enough. After that I do the rest, that one person on the bed could be taken out of the bed a bit later. That’s the way I see things. I can’t create more time than I have.” [Certified nursing assistant]

“If you try to hold on to being done by 10AM and start your break then you will fail. No.. you don’t have to finish by 10. You are not a robot. Our residents aren’t robots. Yes, than you will forget things, skip things or take on other tasks real easy.” [Certified nursing assistant]

### Change is challenging

Participants mention multiple aspects that might influence the absence of intention to stimulate more physical activity. For instance, they acknowledge that their own general well-being might influence their decision-making when providing daily care.

“So in a certain moment… when…. When it’s really busy… and eh.. people lack sleep.. then the staff can get a little grumpy and then we choose… eh… we usually choose safety over freedom.“ [Nurse in training]

Furthermore, the general attitudes of professional caregivers towards change in general and stimulating physical activity is mentioned to have a large influence on the degree of physical activity of the residents on the wards. Participants mention that physical activity in general is an aspect of daily care that is not a priority for professional caregivers and therefore makes it very difficult to incorporate sufficiently.

“Eh.. the… well now I’m going to be very mean. I only work here for a year and the staff is somewhat hospitalized; they are stubborn and stale, they have their own system and they are not going to change it.” [Nurse in training]

“So when it’s about a subject that we all think is important, we usually agree very quickly. But when we talk about smaller adjustments or even tasks that nobody wants to do.. Actually movement is a subject that seems to be neglected a lot of the time.. Those…. The.. Moving, getting people to move is something we neglect. We have to…. It will take a lot of energy.“ [Nurse in training]

### Facilitating and hindering factors to initiate more physical activity in long-term care facilities

As the seven themes describe, professional caregivers view that physical activity is beneficial for the general health and well-being of residents and their individual abilities. This view is an important fundament to implement more physical activity within the physical environment of long-term care facilities. However, improving the degree of physical activity is complex and influenced by various facilitating and hindering factors. Mostly, the inclination to provide safety for residents is of considerable influence, recurrently resulting in the use of physical restraints or discouraging residents to walk. Though, the way daily care is organized is not beneficial for initiating more physical activity. Professional caregivers tend to put the interests of the group over that of the individual residents and do not always feel that it is their responsibility to facilitate physical activity. Lastly, the experienced challenge to realize change within daily long-term care is also an imperative obstruction and influences the overall balance between physical activity and safety (see Fig. 1).

## Discussion

This study aimed to gain more insight into the viewpoints and intentions of professional caregivers on balancing physical activity and safety of people with dementia living in long-term care facilities, from a group perspective. Professional caregivers mention safety and physical activity as important elements in the care for people with dementia. Balancing both is difficult and poses ethical dilemmas. Multiple aspects influence the intentions and behaviour of professional caregivers and play a vital role in the decision making process as it generally tends to shift towards safety over physical activity. A finding supported by earlier research from Robinson et al.(2007) [35].

Many benefits of physical activity for people with dementia are mentioned by professional caregivers within our study. These are focused on maintaining and/or improving general well-being and independency and autonomy. In addition, the benefits for the professional caregivers themselves are mentioned when providing daily care, mainly preserving sufficient mobility and preventing stiffness. Our findings are similar to those of other studies which found that most professional caregivers are well aware of the benefits of physical activity for all people involved in daily care [18]. Especially sustaining independency and the ‘use it or lose it’ principle are widely supported [14].

Our study confirmed that the individual health and abilities of people with dementia in long-term care influence the degree of physical activity within daily care. Some residents lack the mobility to be physically active or lost their intention to move or undertake activities. Apart from physical health, mental health is mentioned to be important, in particular the occurrence of challenging behaviour, which is in general difficult to manage for professional caregivers [36].

Professional caregivers also expressed concerns regarding a phenomenon regularly seen in people with dementia: compulsive walking. Research shows that this behaviour is associated with several negative outcomes [37]. Compulsive walking and other forms of challenging behaviour might influence the way professional caregivers behave and react towards a resident [37]. Therefore, the relation between the resident and the professional caregiver and a personalised approach are essential to gain insight into the reasons for compulsive walking and provide tailored support and care [38]. Our study underlines this point from the perspective of the professional caregivers in dementia care.

The organization of care is mentioned to be important by the professional caregivers. Despite their awareness of the benefits of physical activity for residents and themselves, low staff levels and time restraints influence the incorporation of physical activity in daily care. When experiencing time restraints, professional caregivers mention using shortcuts in daily care in order to make up for lost time. These findings are supported by earlier research [14]. However, even though professional caregivers are aware of the benefits and use shortcuts only when time restraints are experienced, the overall degree of physical activity is still low in long-term care [5–9], which implies that other mechanisms also have an influence.

One of these mechanisms is, according to the findings within this study, the high priority on group interests instead of those of individuals. There is a general believe that it is unacceptable to conduct individual activities with one resident if it results into leaving other colleagues behind to provide daily care for the remaining group of residents. Moreover, another aspect that is mentioned by the professional caregivers within our study is that they do not consider physical activity as their primary responsibility. The existing norm within a group is difficult to break through and requires adequate skills and courage from the particular person or group who addresses them. Care providers in the focus groups mentioned these difficulties. This view is supported by the existing literature, which states that power dynamics are very important when changing social norms. A successful change depends on the view of influential people within the group and the position of the person who wants to realize change within the social hierarchy [39]. It is therefore essential to include key professional caregivers when trying to achieve change within the working culture of a department and to be aware of group dynamics when trying to improve physical activity.

Care teams in long-term (dementia) care are educationally diverse and therefore differ from other health care settings. The potential influence of educational background on safety and physical activity was not mentioned by the participants in this study. Although it is regularly argued that the advanced knowledge and skills of registered nurses (RN) are more beneficial for the overall quality of care, a systematic review of Clemens et al. (2021) states that the results on this topic remain mixed [21]. For instance, no differences are found between CNAs and RNs regarding the use of physical restraints which is strongly linked to physical activity and communication with residents [21]. An important finding of this study is the prioritization of group interests, which may suggest that the group dynamic within care teams is more important than the education or competencies of individual professional caregivers. However, as the job activities of the CNAs are largely care-oriented, they have the largest amount of interaction with residents compared to other professional caregivers and are probably important stakeholders when enhancing physical activity in long-term care [28, 40]. It would be interesting for future studies to further explore this topic.

To the best of our knowledge this is one of the very few studies to provide more insight into the difficulty of balancing between physical activity for individual residents and safety in relation to group interests and group dynamics from the point of view of professional caregivers working in long-term dementia care. Yet, this study also has some weaknesses; as the focus groups were conducted around the change of shift, some time restraints were experienced. Though, the researchers managed to discuss the topics sufficiently. Furthermore, the recruitment of the participants was based on convenience and depended on availability during the specific day. This may have introduced a selection bias. However, all focus groups included sufficient participants with varying backgrounds and years of experience, and the constitution reflected the mix of caregivers in daily practice.

Due to the broad scope of the research topic, it is important for future studies -when building upon these findings- to use different and more extensive research methods. A more holistic understanding of the complex aspects of care addressed in this study, can be obtained by studies with a participatory or ethnographic design. In addition, studies that include more inclusive and objective components will provide the opportunity to approach this topic from a different angle. It will be valuable to connect the current findings on group dynamics and future studies to the existing body of research on physical activity within long-term residential care.  

## Conclusions

Most professional caregivers consider physical activity important for people with dementia living in long-term care facilities. However, due to multiple influencing factors, the balance in long-term care generally tends towards safety over physical activity. Furthermore, in order to stimulate physical activity various limitations are experienced, including the organization of care, the general health of the residents and difficulty to achieve change in daily care. Most importantly, the group interests of both the professional caregivers and the residents have a substantial influence on the incorporation of physical activity in daily care.

## Supplementary Information

**Additional file 1: Appendix 1. **Questions included in interview guide.

## Data Availability

The datasets analysed during the current study are not publicly available due to privacy reasons, but are available from the corresponding author on reasonable request.

## Abbreviations

CAN: Certified Nursing Assistant
RN: Registered Nurse
GPS: Global Positioning System
