# Supplementary material for: ‘’We usually choose safety over freedom’’: results from focus groups with professional caregivers in long-term dementia care
Source: BMC Health Serv Res. 2022 May 20;22:677. doi: 10.1186/s12913-022-07952-0 (PMC9121585; doi:10.1186/s12913-022-07952-0)
Supplement: Supplementary file 1 — Additional file 1: Appendix 1. Questions included in interview guide. [file 12913_2022_7952_MOESM1_ESM.docx]

*Appendix 1: Questions included in interview guide*

| 1. When talking about physical activity on the wards. What comes to mind?  - *What do you consider as physical activity?* - *How is this implemented on the ward?* |
| --- |
| 1. What is your opinion on physical activity for people with dementia living in nursing homes?  - *What do you consider as most important for residents?* - *Is it feasible to achieve this?* - *What challenges do you face?* - *Do you have suggestions and/or solutions to overcome these challenges?* |
| 1. How would the ideal situation look like when it comes to physical activity of residents on the wards?  - *What is needed to achieve this?* - *Who should be involved?* - *Do you see a role for professional caregivers?* |
| 1. When talking about safety on the wards. What comes to mind?  - *Do you consider the ward as safe?* |
| 1. Do you ever face difficult decisions regarding the safety of residents*?*  - *Could you give an example?* |
| 1. What considerations are taken into account during the decision-making regarding the safety of residents?  - *Whom are involved in these decisions?* |
| 1. What is your general view on safety and freedom?  - *Could you come up with an example of a situation wherein you faced an (ethical) dilemma?* - *What do you consider as most important?* - *How do you handle (potential) dilemmas?* |
| 1. What would be the ideal situation for you regarding safety and freedom of residents? |
| 1. Do you see a connection between safety, freedom and physical activity?  - If so, how? - Could you give an example? - How do you handle these situations? |

*Interview guide based on scientific literature (10-14, 16, 18, 35, 36, 41-47) and expert opinion.*

**References**

10. Douma JG, Volkers KM, Engels G, Sonneveld MH, Goossens RHM, Scherder EJA. Setting-related influences on physical inactivity of older adults in residential care settings: a review. BMC Geriatr. 2017;17(1):97.

11. van Alphen HJ, Hortobagyi T, van Heuvelen MJ. Barriers, motivators, and facilitators of physical activity in dementia patients: A systematic review. Arch Gerontol Geriatr. 2016;66:109–18.

12. Galik EM, Resnick B, Pretzer-Aboff I. “Knowing what makes them tick”: motivating cognitively impaired older adults to participate in restorative care. Int J Nurs Pract. 2009;15(1):48–55.

13. den Ouden M, Kuk NO, Zwakhalen SMG, Bleijlevens MHC, Meijers JMM, Hamers JPH. The role of nursing staff in the activities of daily living of nursing home residents. Geriatr Nurs. 2017;38(3):225–30.

14. Benjamin K, Edwards N, Guitard P, Murray MA, Caswell W, Perrier MJ. Factors that influence physical activity in long-term care: perspectives of residents, staff, and significant others. Can J Aging. 2011;30(2):247–58.

16. Ries J. Rehabilitation for Individuals with Dementia: Facilitating Success. Current Geriatrics Reports. 2018;7:59–70.

18. Brett L, Traynor V, Stapley P, Meedya S. Exercise and Dementia in Nursing Homes: Views of Staff and Family Carers. J Aging Phys Act. 2018;26(1):89–96.

35. Robinson L, Hutchings D, Corner L, Finch T, Hughes J, Brittain K, et al. Balancing rights and risks: Conflicting perspectives in the management of wandering in dementia. Health Risk Soc. 2007;9(4):389–406.

36. Evripidou M, Charalambous A, Middleton N, Papastavrou E. Nurses’ knowledge and attitudes about dementia care: Systematic literature review. Perspect Psychiatr Care. 2019;55(1):48–60.

41. Evans EA, Perkins E, Clarke P, Haines A, Baldwin A, Whittington R. Care home manager attitudes to balancing risk and autonomy for residents with dementia. Aging Ment Health. 2018;22(2):261–9.

42. Fitzgerald TG, Hadjistavropoulos T, MacNab YC. Caregiver fear of falling and functional ability among seniors residing in long-term care facilities. Gerontology. 2009;55(4):460–7.

43. Koren MJ. Person-centered care for nursing home residents: the culture-change

movement. Health affairs (Project Hope). 2010;29(2):312–7.

44. Low LF, Fletcher J, Goodenough B, Jeon YH, Etherton-Beer C, MacAndrew M, et al. A Systematic Review of Interventions to Change Staff Care Practices in Order to Improve Resident Outcomes in Nursing Homes. PLoS One. 2015;10(11):e0140711.

45. Mitchell G, Agnelli J. Person-centred care for people with dementia: Kitwood reconsidered. Nursing standard. 2015;30(7):46–50.

46. Davison TE, Camões-Costa V, Clark A. Adjusting to life in a residential aged care facility: Perspectives of people with dementia, family members and facility care staff. J Clin Nurs. 2019;28(21–22):3901–13.

47. McGreevy J. Implementing culture change in long-term dementia care settings. Nursing standard. 2016;30(19):44–50.
